# Prognostic value of tumoral and peritumoral magnetic resonance parameters in osteosarcoma patients for monitoring chemotherapy response

**DOI:** 10.1007/s00330-020-07338-y

**Published:** 2020-11-04

**Authors:** Yuewen Hao, Rui An, Yingsen Xue, Fan Li, Hong Wang, Jianmin Zheng, Linni Fan, Jixin Liu, Hongbin Fan, Hong Yin

**Affiliations:** 1 Department of Radiology, Xi’an Children’s Hospital, Xi’an, Shaanxi China; 2 Department of Radiology, Xijing Hospital, Fourth Military Medical University, Xi’an, Shaanxi China; 3 Department of Orthopaedic Surgery, Northwest Women’s and Children’s Hospital, Xi’an, Shaanxi China; 4 Department of Health Statistics, Fourth Military Medical University, Xi’an, Shaanxi China; 5 Department of Pathology, Fourth Military Medical University, Xi’an, Shaanxi China; 6 School of Life Science and Technology, Xidian University, Xi’an, Shaanxi China; 7 Department of Orthopaedic Surgery, Xijing Hospital, Fourth Military Medical University, Xi’an, Shaanxi China

**Keywords:** Osteosarcoma, Magnetic resonance imaging, Chemotherapy

## Abstract

**Objectives:**

To evaluate parameters of diffusion-weighted imaging (DWI) and dynamic contrast-enhanced magnetic resonance imaging (DCE-MRI) as early imaging indicators of tumor histologic response to pre-operative neoadjuvant chemotherapy and as probable prognostic factors for event-free survival (EFS) and overall survival in osteosarcoma (OS) in both tumoral and peritumoral areas.

**Methods:**

Thirty-four OS patients who received three courses of neoadjuvant chemotherapy followed by surgery during 2014–2018 were enrolled in this study. All patients underwent baseline and post-chemotherapy DWI and DCE-MRI. Lesion region was defined as the tumoral area and peritumoral area. Parameters of apparent diffusion coefficient, capacity transfer constant (Ktrans), elimination rate constant, extravascular extracellular space volume ratio (Ve), and initial area under the curve as well as corresponding differences between pre- and post-chemotherapy in lesion regions were evaluated. Receiver operating characteristic analysis was used to evaluate the diagnostic performance of these parameters. The associations of all parameters with tumor histologic response, EFS, and overall survival were also calculated.

**Results:**

In the tumor area, moderate evidence was found that post-Ktrans was lower in responders as compared with that in poor responders (*p* = 0.04, false discovery rate [FDR] corrected), and ΔKtrans exhibited significant between-groups differences (*p* = 0.04, Bonferroni corrected; or *p* = 0.006, FDR corrected). Weak evidence for the between-groups difference was found in the Ve in the peritumoral area (*p* = 0.025 before treatment and *p* = 0.021 after treatment, uncorrected). Furthermore, lower post-Ktrans in the tumoral area and lower pre-Ve in the peritumoral area were significant prognostic indicators for longer EFS (*p* = 0.002, *p* = 0.026) and overall survival (*p* = 0.003, *p* = 0.023).

**Conclusions:**

In OS, DWI and DCE-MRI parameters in both tumoral and peritumoral areas can reflect the chemotherapy response and prognosticate EFS and overall survival.

**Key Points:**

*• Peritumoral MRI parameters can reflect the chemotherapy response in OS patients.*

*• Peritumoral MRI parameters can predict EFS and overall survival in OS patients.*

*• MRI parameters may be predictive factors for evaluating chemotherapy efficacy and EFS.*

## Introduction

Osteosarcoma (OS) is the most common malignant bone tumor with peak incidence in children and adolescents [1]. The current treatment strategy for OS is neoadjuvant chemotherapy that induces tumor necrosis to facilitate surgical resection. The combination of pre-operative and post-operative chemotherapy improved the long-term survival rate from 20 to nearly 70% in patients compared with surgery alone [2–6].

In OS, chemotherapy-induced necrosis is a well-accepted predictive factor for prognosis. Post-chemotherapy tumor necrosis in more than 90% cases is associated with significantly higher survival rate [7, 8]. However, as necrosis can only be assessed after tumor resection post-chemotherapy, it may not represent a true early prognostic factor. Thus, it would be desirable to have a non-invasive method to predict clinical response to neoadjuvant chemotherapy before surgical resection.

Magnetic resonance imaging (MRI) has been developed to be the most important method for primary bone tumor diagnosis and post-operative tumor relapse detection. Among different MR measurements, dynamic contrast-enhanced MR imaging (DCE-MRI) (with its ability to measure tissue microvasculature properties such as tissue perfusion, capillary permeability, and interstitial volume [9, 10]) and diffusion-weighted (DW)-MRI (with its ability to assess impedance of water molecule diffusion, which is most dependent on the tissue cellularity [11–13]) have become the commonly used modalities in OS assessment. The change of capacity transfer constant (Ktrans) in DCE-MRI and apparent diffusion coefficient (ADC) in DW-MRI post-chemotherapy were correlated with pathological response in previous studies [14–20]. However, no parameter has been verified as a prognostic factor for patient outcomes in OS. Moreover, all previous studies focused only on the image parameter changes in the tumoral area, and the area adjacent to tumor was neglected. Slaughter et al suggested that the residual “alterated field” adjacent to the tumor could be the leading cause of treatment failure and local recurrence [21]. Several fundamental studies have demonstrated the association between tumor local recurrence and cancer-related gene expression in peritumoral area; however, whether clinical diagnostic imaging is correlated with tumor progression and treatment effect remains unclear, as it was hypothesized that parameters in peritumoral area detected by MRI would be possible indicators of response or prognostic factors for patient outcome of neoadjuvant chemotherapy.

In this study, we investigated the diagnostic and prognostic value of DW-MRI and DCE-MRI parameters in post-chemotherapy OS patients in both tumoral and peritumoral areas.

## Materials and methods

### Patients and treatment

Thirty-four patients with high-grade non-metastatic and potentially resectable OS (median: 14 years, range 6–29 years; male = 18, female = 16) were enrolled in this study from January 2014 to June 2018 in the orthopedics department of Xijing Hospital, Fourth Military Medical University. Exclusion criteria were inability to give informed consent, history of surgical resection, radiotherapy or chemotherapy, non-lower extremity OS, and contraindications to MR or MR contrast media. Two patients deemed to be ineligible after enrollment were also excluded, resulting in a total of 34 patients in the study. This retrospective diagnostic study was approved by the institutional review committee of our center.

**Fig. 1:**
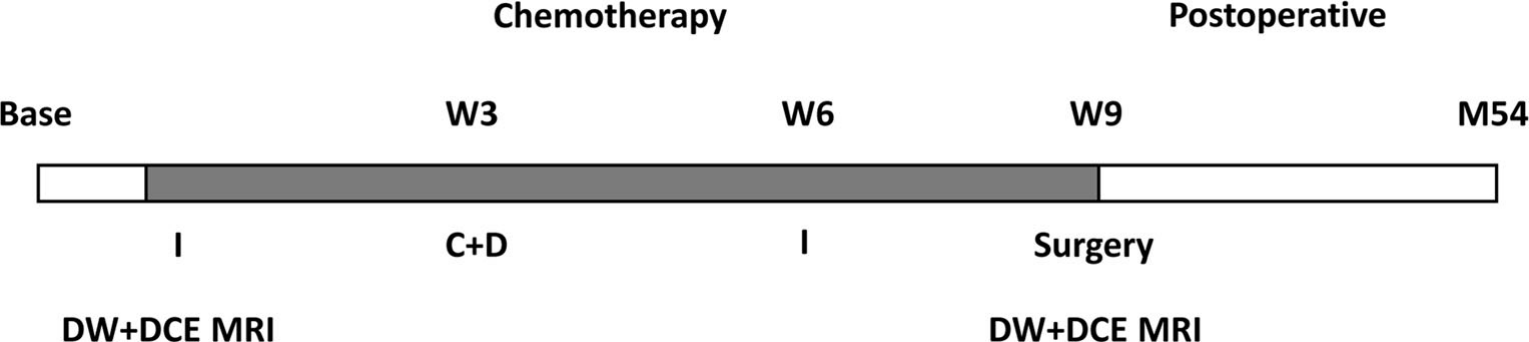
Treatment schedule and timing of DW- and DCE-MR imaging. Base represents the baseline before treatment. I, ifosfamide; C, cisplatin; D, doxorubicin; W, week; M, month

All patients received three courses of neoadjuvant chemotherapy before operation: ifosfamide (12 g/m^2^) at week 9, cisplatin (60 g/m^2^) and doxorubicin (120 mg/m^2^) at week 6, and ifosfamide (12 g/m2) at week 3 before surgical resection. The treatment and imaging schedules are shown in Fig. 1.

### MRI data acquisition

In this study, a 1.5-T MRI system (Siemens Healthineers) was used. All patients underwent MRI examinations twice including DCE-MRI and multi–*b* value high-resolution diffusion-weighted imaging examination at week 0 (before neoadjuvant therapy) and week 9 (before tumor resection/post-neoadjuvant therapy), respectively. A standard examination was performed including coronary and transverse fat-saturated T_1_WI and T_2_WI.

For DCE-MRI protocols, firstly the T1 map was acquired by using the VFA method (variable flip angle technique) with the unenhanced T1-weighted VIBE (volumetric interpolated breath-hold examination) sequence, with flip angles of 2° and 15°, which was automatically calculated by the Tissue 4D software (Siemens Healthineers) at the workstation. The patient was given injections of 0.1 mmol/kg of gadodiamide at a rate of 4 ml/s followed by a saline flush. Dynamic imaging was performed using the VIBE sequence with the parameters as follows: TR/TE = 3.79/1.35 ms, slice thickness = 3.5 mm, acquisition matrix = 340 × 244; the total acquisition time was almost 6 min for 60 measurements. Multi–*b* value high-resolution DWI examination was performed with the RESOLVE sequence (readout segmentation of long variable echo-trains), and the ADC value was generated automatically after scanning. The details of scan parameters are as follows: TR = 2000 ms, TE1 = 59 ms, TE2 = 95 ms, 150° flip angle, matrix = 360 × 288, slice thickness = 5 mm, readout segments = 7, echo spacing = 0.38 ms, *b* value = 0, 50, 100, 150, 200, 300, 500, 800 s/mm^2^; totally scan time was 6.16 min.

### Evaluation of response and survival

After definitive surgery at week 9, histologic response was assessed by experienced pathologists using the four-grade system of Huvos [2, 4]. Tumor necrosis was graded as grade I (0–49%), grade II (50–89%), grade III (90–99%), and grade IV (100%). Patients with grades III and IV necrosis (≥ 90% necrosis) are considered good responders (responder group) and those with grades I and II necrosis (< 90% necrosis) as poor responders (poor-responder group).

For metastatic disease is an overwhelming factor in predicating survival, only patients with localized disease can be included in the analyses of event-free survival (EFS) and overall survival. EFS was defined as the time interval from the date of study enrollment to the date of the first event (disease progression, relapse, secondary malignancy, or death) or to the date of last follow-up for patients without events. Overall survival was defined as the time from the date of study enrollment to the date of death from any cause or to the last follow-up date.

### DCE-MRI analysis

DCE-MRI raw dates were uploaded to the Siemens post-processing station, and the Tissue 4D software was used to analyze DCE data. Tumor delineation was performed by two pediatric radiologists (clinical experience of more than 10 years) using an interactive display to select the region of interest (ROI) of the tumoral area on each slice that ensured that tumor boundary selection was consistent across all time points. The adjacent area of the ROI (peritumoral area/molecular boundary) refers to the periphery area within 2 cm from the soft tissue mass (tumoral area). All measurements were kept away from the vessels to avoid interference of parameters.

Five tumoral areas were randomly delineated on the axis of ADC map and DCE map as ROI. The tumor region and peritumoral region of each layer were manually delineated, and the average ADC value was calculated.

Firstly, the motion correction of the images in each period was carried out; secondly, T1 mapping images were automatically registered; thirdly, by referring to the two-compartment pharmacokinetic model, AIF (arterial input function)-slow was fitted with TIC (time of intensity curve) type to generate false color images; fourthly, for each pixel inside the tumor region and peritumoral region ROI, the four quantitative parameters, Ktrans, elimination rate constant (Kep), extravascular extracellular space volume ratio (Ve), and IAUC, were computed.

The corresponding differences (Δ means parameter post-chemotherapy minus pre-chemotherapy, e.g., ΔKtrans = post-Ktrans − pre-Ktrans) of each averaged kinetic parameter between pre- and post-neoadjuvant therapy of tumoral and peritumoral ROI were also computed for further statistical analysis. All parameters were used for assessing neoadjuvant therapy response, EFS, and overall survival.

### HE staining

In this study, 34 patients underwent HE staining before and after chemotherapy. Paraffin sections were immersed in xylene for dewaxing for 15 min, hematoxylin staining was performed for 2 min, 1% hydrochloric acid alcohol for 3 min, water washing tablets, gradient alcohol for gradual dehydration, eosin staining for 2 min, and neutral gum sealing piece.

### Statistical analysis

Non-parametric Wilcoxon’s signed-rank tests were used to examine the difference of ADC and eight DCE-MRI parameters (Ktrans, Kep, Ve, AUC, ΔKtrans, ΔKep, ΔVe, and ΔAUC in the ROI were determined for patients at two time points (week 0 [pre-chemotherapy] and week 9 [post-chemotherapy])). Mann-Whitney tests were used to examine the difference of each parameter between responders and poor responders. Bonferroni’s and false discovery rate corrections were used for multiple comparison corrections. Receiver operating characteristic (ROC) curves were generated to compare parameters for differentiating responders and poor responders. Area under the curve, standard error, 95% confidence interval (95% CI), and optimal cutoff were estimated in the ROC analyses.

Univariate and multivariate analyses of EFS/overall survival at different areas (tumor or peritumoral area) were performed utilizing Cox proportional hazard model to explore associations between outcomes (EFS and overall survival) with each parameter, to adjust for potential confounding variables. Only covariates that were associated with the risk of primary study endpoint at univariate analysis with *p* < 0.10 were then included in the multivariate analysis. Selection of variables included in the multivariate model was done with backward elimination based on covariates that were significantly associated with the primary efficacy endpoint (*p* > 0.10 for exclusion). Then, patients were categorized into two groups using the significant median parameter values as cut-points. EFS and overall survival distributions were estimated using the method of Kaplan and Meier, and differences were examined using the log-rank test.

All the statistical analyses were performed using the Prism 6.02 and SAS software (version 9.1); two side *p* values < 0.05 were considered statistically significant.

## Results

**Fig. 2:**
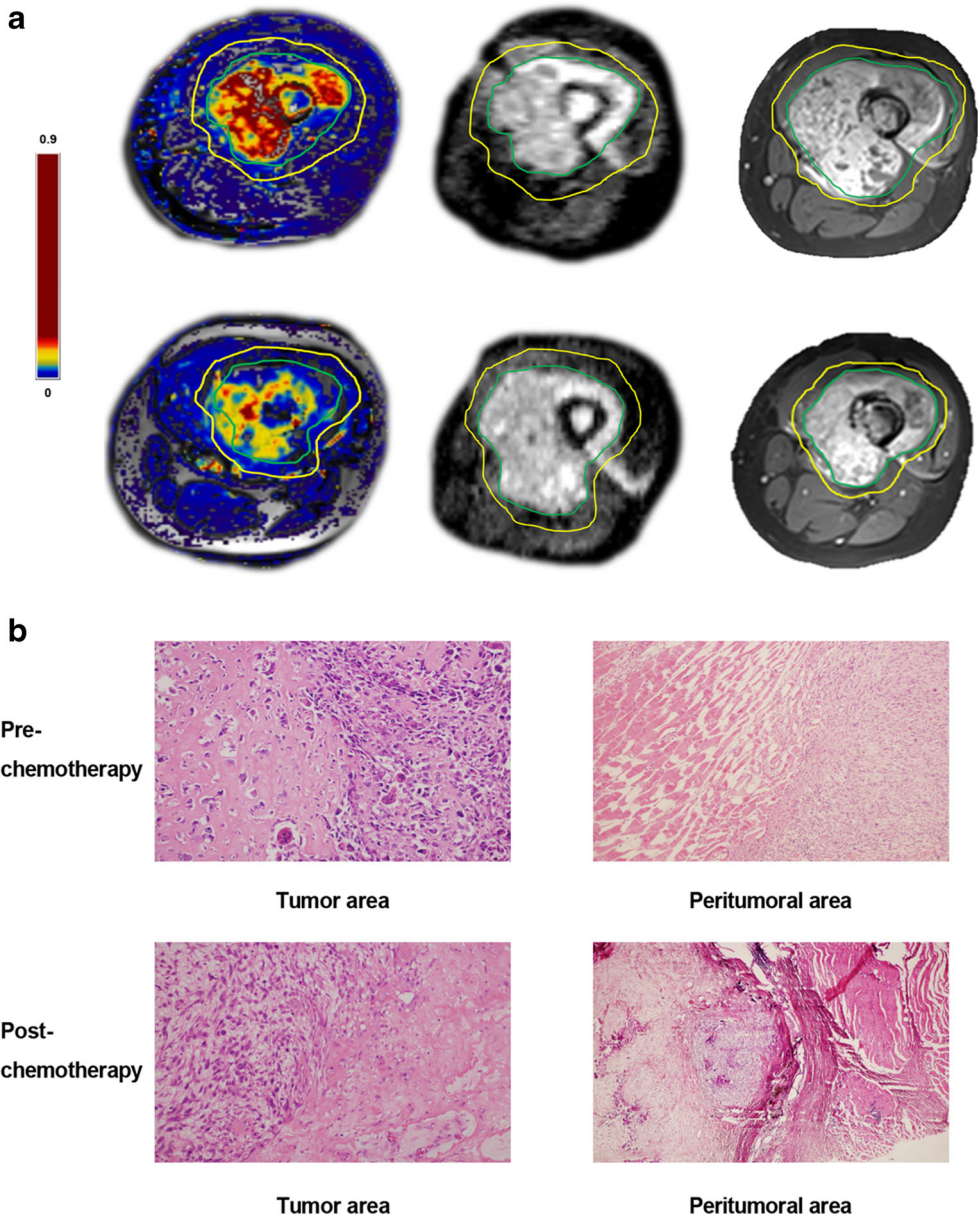
**a** The contrast-enhanced image, DCE-MR map (Ktrans as example), and ADC map in baseline examination (upper row) and post-chemotherapy (lower row) are displayed from left to right. The green line is the boundary of tumor ROI, and the yellow line is the boundary of peritumoral ROI drawn by a radiologist. The color bar is displayed on the left. **b** Picture 1, morphological observation in the tumor area before chemotherapy by HE staining (original magnification: × 100). Picture 2, morphological observation in the tumor area after chemotherapy. Picture 3, morphological observation in the peritumoral area before chemotherapy. Picture 4, morphological observation in the peritumoral area after chemotherapy.

A total of 34 patients were available for analysis in the study. The responder group consisted of 19 patients (55.9%) and the poor-responder group of 15 patients (44.1%) (shown in Table 1). All 34 patients underwent surgery; in 26 patients, limb salvage surgery was performed while the other 8 patients underwent amputation. ROI of tumoral and peritumoral areas were delineated by radiologists with computer software as shown in Fig. 2a. All results for tumoral and peritumoral areas were compared.

**Table 1:** Patient characteristics

Variable	Number of patients (n = 34)
Gender (male: female)	18:16
Age	Years
Median age (range)	14 (6–29)
Mean age ± SD	15 ± 5.4
Primary sites	Number of patients (%)
Upper end of tibia	14 (41%)
Lower end of femur	20 (59%)
Pathological grade	III–IV
Metastasis	0 (0)
Tumor necrosis rate	
TNR > 90%	19
TNR < 90%	15

*SD* standard deviation

### Imaging and histologic response

First, we compared the parameters of pre- (week 0) and post-neoadjuvant therapy (week 9) between groups (Table 2). In the tumoral area, post-Ktrans of the responder group was lower than that of the poor-responder group (*p* = 0.04, FDR corrected), and ΔKtrans exhibited a significant between-groups difference (*p* = 0.04, Bonferroni corrected; or *p* = 0.006, FDR corrected). In the peritumoral area, post-ADC of the responder group was higher than that of the poor-responder group (*p* = 0.01, FDR corrected), and ΔADC exhibited a significant between-groups difference (*p* = 0.008, FDR corrected). Additionally, weak evidence for the between-groups differences was also found in the Ve at different time points (*p* < 0.05, uncorrected).

**Table 2:** Comparison between groups at different time points

	Pre-chemotherapy		*p*1	*p*2	*p*3	Post-chemotherapy		*p*1	*p*2	*p*3	Difference between post-chemotherapy and pre-chemotherapy		*p*1	*p*2	*p*3
	Responder group	Poor-responder group				Responder group	Poor-responder group				Responder group	Poor-responder group			
Tumor area															
Ktrans/min	0.47 (0.39, 0.55)	0.59 (0.27, 0.72)	NS	NS	NS	0.21 (0.15, 0.23)	0.40 (0.23, 0.57)	0.009	NS	0.04	− 0.24 (− 0.31, − 0.20)	− 0.065 (− 0.2, − 0.042)	0.0007	0.04	0.006
Kep/min	2.03 (1.27, 2.646)	1.54 (0.86, 2.61)	NS	NS	NS	1.47 (1.03, 2.23)	1.37 (1.056, 2.963)	NS	NS	NS	− 0.51 (− 0.65, 0.21)	0.25 (− 0.08, 0.55)	NS	NS	NS
Ve	0.35 (0.22, 0.43)	0.36 (0.28, 0.60)	NS	NS	NS	0.33 (0.23, 0.47)	0.43 (0.22, 0.58)	NS	NS	NS	− 0.016 (− 0.11, 0.18)	− 0.041 (− 0.13, 0.11)	NS	NS	NS
IAUC	8.15 (5.94, 19.56)	8.96 (6.42, 10.56)	NS	NS	NS	6.76 (5.22, 9.00)	7.29 (5.68, 10.21)	NS	NS	NS	− 0.72 (− 3.23, 0.97)	−0.84(−2.7,0.30)	NS	NS	NS
ADC (× 10^−3^)	1.08 (0.89, 1.33)	1.35 (1.18, 1.48)	0.001	0.05	0.006	1.68 (1.35, 2.01)	1.65 (1.40, 1.77)	NS	NS	NS	0.73 (0.32, 1.0)	0.33 (0.24, 0.42)	0.015	NS	NS
Peritumoral area															
Ktrans/min	0.26 (0.21, 0.33)	0.36 (0.18, 0.46)	NS	NS	NS	0.13 (0.10, 0.17)	0.20 (0.11, 0.32)	NS	NS	NS	− 0.14 (− 0.19, − 0.10)	− 0.09 (− 0.17, − 0.04)	0.04	NS	NS
Kep/min	1.49 (1.17, 1.97)	0.75 (0.59, 1.97)	0.037	NS	NS	1.57 (0.74, 3.09)	1.39 (0.53, 1.60)	NS	NS	NS	0.26 (− 0.75, 1.38)	0.12 (− 0.52, 0.91)	NS	NS	NS
Ve	0.23 (0.15, 0.26)	0.38 (0.21, 0.75)	0.025	NS	NS	0.12 (0.07, 0.26)	0.27 (0.26, 0.29)	0.021	NS	NS	− 0.066 (− 0.13, 0.031)	− 0.08 (− 0.46, − 0.002)	NS	NS	NS
IAUC	7.0 (2.53, 10.33)	5.90 (4.88, 12.42)	NS	NS	NS	5.64 (3.92, 8.35)	7.51 (3.86, 9.41)	0.028	NS	NS	− 2.12 (− 3.64, − 0.56)	− 1.91 (− 3.37, − 0.39)	NS	NS	NS
ADC (× 10^−3^)	1.35 (1.04, 1.54)	1.66 (1.33, 1.81)	NS	NS	NS	2.35 (2.15, 2.51)	1.98 (1.86, 2.09)	0.002	0.1	0.01	0.89 (0.67, 1.31)	0.26 (0.13, 0.45)	0.0015	0.075	0.008

Non-parametric Wilcoxon’s signed-rank tests were used to examine the difference of ADC and eight DCE-MRI parameters. Mann-Whitney tests were used to examine the difference of each parameter between groups. Multiple comparisons (50 times) were performed. *p*1: uncorrection; *p*2: Bonferroni’s correction; *p*3: false discovery rate correction. *NS* non-significant

Second, we measured the pre- and post-chemotherapy parameters in two groups with the self-contrasted method (Table 3). In the responder group, Ktrans and ADC exhibited significant changes both in the tumoral area and in the peritumoral area (*p* < 0.05, corrected). In the poor-responder group, only ADC showed significant changes both in the tumoral area and in the peritumoral area (*p* < 0.05, corrected).

**Table 3:** Comparison between different time points

	Responder group		*p*1	*p2*	*p*3	Poor-responder group		*p*1	*p*2	*p*3
	Pre-chemotherapy	Post-chemotherapy				Pre-chemotherapy	Post-chemotherapy			
Tumor area										
Ktrans/min	0.47 (0.39, 0.55)	0.21 (0.15, 0.23)	0.0006	0.03	0.006	0.592 (0.267, 0.718)	0.395 (0.228, 0.567)	NS	NS	NS
Kep/min	2.03 (1.27, 2.64)	1.46 (1.03, 2.23)	NS	NS	NS	1.544 (0.856, 2.610)	1.371 (1.056, 2.963)	NS	NS	NS
Ve	0.35 (0.22, 0.43)	0.33 (0.23, 0.47)	NS	NS	NS	0.360 (0.278, 0.602)	0.428 (0.222, 0.582)	NS	NS	NS
IAUC	8.15 (5.94, 19.6)	6.76 (5.22, 8.99)	NS	NS	NS	8.962 (6.422, 10.560)	7.293 (5.684, 10.210)	NS	NS	NS
ADC (× 10^−3^)	1.08 (0.89, 1.33)	1.68 (1.35, 2.01)	0.000002	0.0001	0.0001	1.350 (1.180, 1.480)	1.650 (1.400, 1.770)	0.0002	0.001	0.003
Peritumoral area										
Ktrans/min	0.26 (0.21, 0.33)	0.13 (0.10, 0.17)	0.0001	0.006	0.002	0.362 (0.178, 0.464)	0.199 (0.109, 0.316)	NS	NS	NS
Kep/min	1.49 (1.17, 1.97)	1.57 (0.74, 3.09)	NS	NS	NS	0.753 (0.587, 1.969)	1.391 (0.534, 1.596)	NS	NS	NS
Ve	0.23 (0.15, 0.26)	0.12 (0.07, 0.26)	NS	NS	NS	0.377 (0.206, 0.751)	0.269 (0.257, 0.291)	NS	NS	NS
IAUC	7.01 (2.53, 10.33)	5.64 (3.92, 8.35)	NS	NS	NS	5.945 (4.882, 12.420)	7.511 (3.857, 9.406)	NS	NS	NS
ADC (× 10^−3^)	1.35 (1.04, 1.54)	2.35 (2.15, 2.51)	0.00004	0.002	0.001	1.655 (1.325, 1.810)	1.980 (1.858, 2.085)	0.0008	0.04	0.006

Non-parametric Wilcoxon’s signed-rank tests were used to examine the difference of ADC and eight DCE-MRI parameters. Mann-Whitney tests were used to examine the difference of each parameter between different time points. Multiple comparisons (50 times) were performed. *p*1: uncorrection; *p*2: Bonferroni’s correction; *p*3: false discovery rate correction. *NS* non-significant

### HE staining of tumoral and peritumoral areas

The results of the tumoral area showed that the heteromorphic cells were oval or short fusiform; epithelioid and transparent cells were arranged in sheets and nests, with ribbon-like bone matrix, hyperchromatic nuclei, mitotic figure, partial bone trabecular degeneration and calcification, and proliferation of vascular fiber tissue. Gross pathological specimens were taken after the surgery and stained with HE after chemotherapy, large areas of necrotic cells were observed under the microscope, and few tumor cells survived. The results of HE staining showed that normal muscle tissue could be seen outside the tumor cells before chemotherapy. After chemotherapy, large areas of necrotic cells were observed under the microscope in the tumoral area, and few tumor cells survived, with a little calcification and pigmentation. A small number of muscle cells were seen in the para-tumoral area, and no obvious signs of tumor cell infiltration were observed.

### Comparison of ROC curves

We generated best threshold values for above-mentioned significant different MRI parameters as determined by the ROC curves. The sensitivity and specificity of the respective parameters were also measured at each threshold value to predict good histological response.

### Imaging and event-free and overall survival

**Fig. 3:**
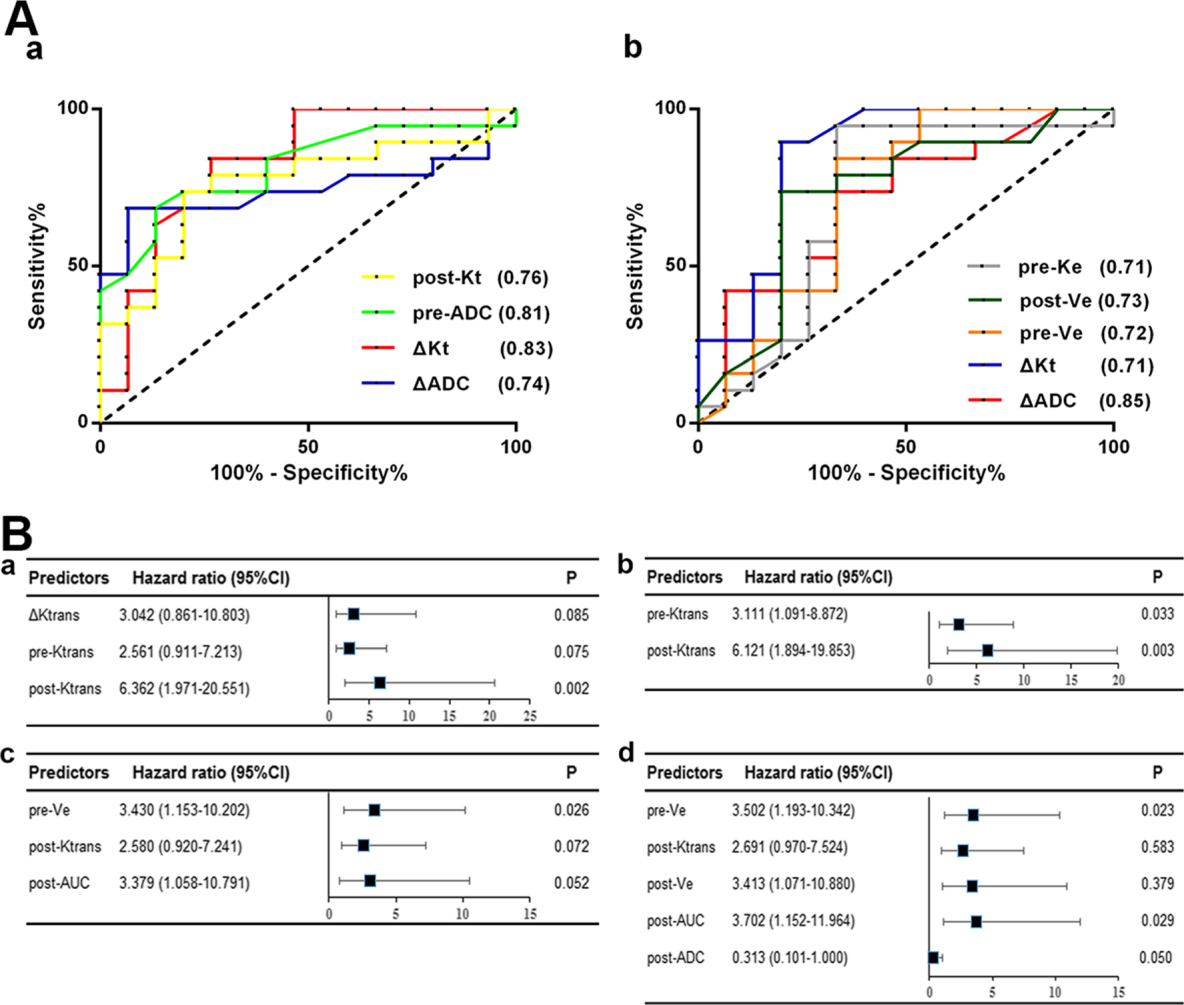
**A** ROC curve for parameters in tumor and peritumoral areas. (**a**) tumor area; (**b**) peritumoral area. **B** Cox hazard models were used to estimate the hazard ratios and 95% confidence intervals of DW- and DCE-MRI parameters in association with EFS/overall survival. CI, confidence interval

**Fig. 4:**
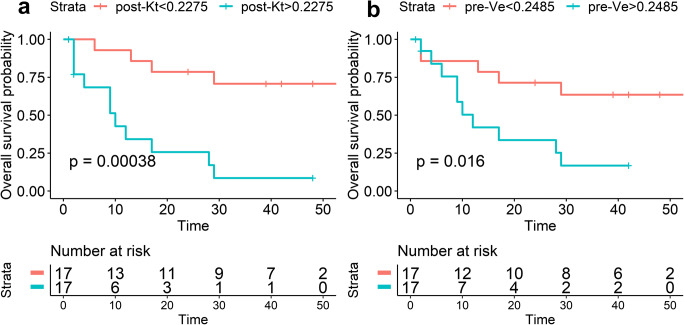
Event-free survival curves for subgroups stratified by the median value of post-Kt in the tumor area (**a**) and pre-Ve in the peritumoral area (**b**). *p* values were obtained from exact log-rank tests

A Cox proportional hazards model was used to analyze the associations between all MR parameters and EFS/overall survival. As shown in Fig. 3a, in the tumoral area, lower post-Ktrans was significantly associated with longer EFS (*p* < 0.01). In peritumoral area, lower pre-Ve (pre-neoadjuvant therapy/week 0) was significantly associated with longer EFS (*p* = 0.03). Moreover, analysis of the average post-Ktrans in the tumoral area as well as pre-Ve in the peritumoral area was performed using Kaplan-Meier curves stratified according to the median values for the cohort to explore the prognostic effect of these parameters. As shown in Fig. 4, patients with lower values of tumor post-Ktrans had a significant higher EFS rate (*p* < 0.001). Patients with lower values of peritumoral pre-Ve had a significant higher EFS rate (*p* = 0.017).

**Fig. 5:**
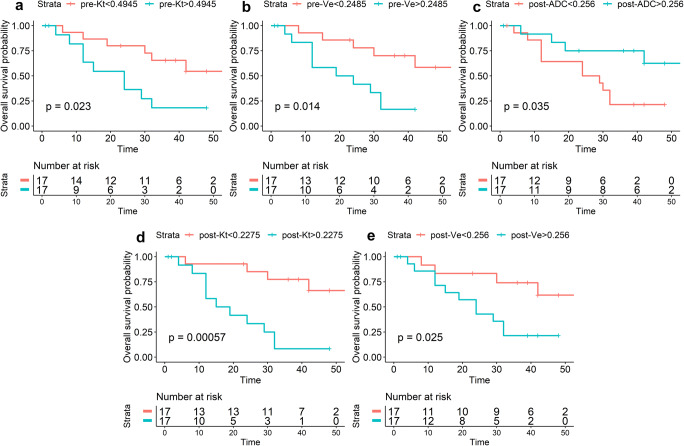
Overall survival curves for subgroups stratified by the median value of pre-Kt (**a**) and post-Kt (**b**) in the tumor area; stratified by the median value of pre-Ve (**c**), post-Ve (**d**), and post-ADC (**e**) in the peritumoral area. *p* values were obtained from exact log-rank tests

Overall survival was also analyzed with the same method. As shown in Fig. 5, in the tumoral area, lower pre- and post-Ktrans were significantly associated with longer OS (*p* = 0.023, *p* < 0.001). In the peritumoral area, lower pre- and post-Ve were significantly associated with longer OS (*p* = 0.014, *p* = 0.025), and higher post-ADC was significantly associated with longer OS (*p* = 0.036).

## Discussion

In this study, DW- and DCE-MRI parameters in both tumoral and peritumoral areas were analyzed to explore their relationships with neoadjuvant therapy outcomes in OS patients. First, Ktrans was significantly decreased and ADC was significantly increased post-chemotherapy in responders and poor responders in both areas. Second, ΔKtrans and ΔADC were significantly different between groups in both the areas and well correlated with histologic response. Third, post-Ktrans in the tumoral area and pre-Ve in the peritumoral area were significant prognostic factors for both EFS and overall survival.

OS, with peak incidence in children and adolescents, is the most common primary malignancy of bone. The main cause of mortality in patients with OS is lung metastasis, and approximately 10~20% of patients are diagnosed with metastatic OS upon identification of the disease in the USA [22]. Prognosis for OS patients has greatly improved since the introduction of effective chemotherapeutic agents in the 1970s and subsequent developments in neoadjuvant chemotherapy. The chemotherapy regimen that was adopted in this study was mentioned previously as most frequently used in our orthopedics department.

The treatment response of OS is commonly verified by the necrosis rate with pathological examination after tumor resection which is the last procedure in the treatment process; thus, imaging diagnosis such as MR imaging was urgently needed to replace the lagged indicator for early evaluation response. DW and DCE-MRI were the most frequently used in tumor diagnosing with their own typical features, reflecting tumor characteristics. DW-MRI well reflected the membrane permeability between intra- and extracellular compartments with the surrogate marker of water diffusion, and DCE-MRI better indicated the leaky newly formed tumor capillaries, reflected by the contrast medium uptake capacity. Several studies have reported that ADC changes are greater in osteogenic patients with higher necrosis rate (> 90%) than in those with low necrosis rate (< 90%) post-chemotherapy [23], and lower post-chemotherapy Ktrans was associated with greater necrosis percent and longer event-free survival in OS patients [24]. Consistent with other studies, all patients in week 9 (post-chemotherapy) had higher ADC and lower Ktrans values than in week 0 (pre-chemotherapy), which means tumors were directly affected by chemotherapy drugs. Subtle changes in the degree of restriction of diffusion such as an alteration in cell membrane integrity or permeability to water, are reflected in changes in the DW signal [25]. In our study, in the tumoral area, ΔADC in the responder group was significantly higher than in the poor-responder group, and the higher necrosis rate reflected lower restriction of water molecule motion. ΔKtrans in the responder group was also significantly higher than that in the poor-responder group; similarly, larger necrosis indicated decreased capillary permeability and reflected better response. Correspondingly, parameters in the peritumoral area had similar ΔKtrans and ΔADC variation trend. ROC curves indicated that among all parameters in both tumor and peritumoral areas that reflected histologic response, ΔKtrans in the tumoral area and ΔADC in the peritumoral area had the best diagnostic values.

MRI parameters can potentially reflect early changes in treatment especially in patients who resist or are unable to receive surgery for histologic response assessment. Currently, the existence of metastasis or not is the key prognostic factor that determines the treatment protocols for OS, and there is a pressing need to identify prognostic factors especially for the majority of patients who have localized disease. The prognostic significance of the DW- and DCE-MRI parameters will be useful to stratify patients in clinical trials and to identify the group of patients with private customized protocols.

In this study, it was found that no tumor cells were observed in the peritumoral area before and after chemotherapy by HE staining, but the targeted genes or specific molecules in the edema zone were over-expressed to varying degrees, and their biological behaviors were also changed. We hypothesized that peritumoral capillary permeability would change; in fact, the research finding showed that DCE-MRI parameters had changed before and after neoadjuvant chemotherapy, which was the imaging basis of DCE-MRI.

The DCE-MRI parameters have been shown to be associated with clinical outcome manifested as histologic response and survival in some trials. Reddick et al found that lower Kep post-neoadjuvant therapy was significantly predictive of EFS [26]. This is very similar to our results in which a lower post-Ktrans in the tumoral area was significantly associated with better EFS. Several studies have demonstrated that Ktrans was positively correlated with the tumors’ malignancy grade, where neo-formed leaky tumor capillaries can determine a rapid gadolinium uptake and an early wash-out as well as overall shorter first pass, especially when compared with healthy tissues [27–29]. It should be noted that the associations between DW/DCE-MRI parameters and histologic response may not be equated with associations between the same DW/DCE-MRI parameters and EFS (or overall survival). All response or prognostic indicators must be verified independently [30]. In the current study, lower pre- and post-Ktrans in the tumoral area were significantly associated with better overall survival.

To our knowledge, it is one of the first studies to identify the diagnostic imaging values in peritumoral areas. Although few studies had reported the relationship between the tumoral area MR imaging and EFS or overall survival, we focused the adjacent area to supplement the understanding of MR imaging in predicting prognosis. Meanwhile, parameters in the peritumoral area may be better in consistency compared with the tumoral area that may be influenced by complex circumstances such as necrosis, hemorrhage, edema, and newly formed capillaries. Slaughter et al hypothesized that a residual “alterated field” in the area adjacent to the tumor could be the leading cause of treatment failure and local recurrence [21]. Several studies emphasized on the relationships between molecular marker and therapeutic effect and recurrence [31], and the silencing of cancer-related genes via epigenetic alterations which have been shown to play an important role in tumor progression and to be an early event in the carcinogenesis process [32]. We focused on the diagnostic imaging difference in the peritumoral area, hoping to offer effective suggestions in the clinical treatment of OS. All parameters were independently analyzed and the results showed that lower pre-Ve instead of post-Ktrans was significantly associated with better EFS, and lower pre- and post-Ve were significantly predictive of better overall survival. In Guo et al’s study, the tumoral area was defined as outer and inner halves; the results showed that the difference of Ve between the outer and inner halves of tumor at baseline (pre-treatment) was significantly associated with the EFS. Although the definition of parameters was different (they divided the tumoral area as outer and inner halves, and we defined the peritumoral area within 2 cm around the tumor boundary), the results may have implications that peritumoral pre-Ve (consistent with their Ve at baseline) in our study to some extent is similar with the outer minus inner Ve, and the variation trend may change from peritumoral area to tumor core, which needs further confirmation. Moreover, higher post-ADC in peritumoral area was marginally associated with better overall survival. Recently, one similar study reported that the tumor surface regularity was a predictor of survival for patients who underwent complete resection in glioblastoma patients, indirectly illustrating the significance of peritumoral area [33].

There are some limitations to this study. First is the single-centered study and limited case number. With the number of patients 34, two parameters were marginally significant that may need enlarged patient number. Second, peritumoral pathological sequencing should be performed to further demonstrate the correlation between imaging parameters and gene variations. Third, chemotherapy regimen was fixed; results can only prove the relationship between imaging parameters and chemotherapy response in such specified condition.

## Conclusion

In conclusion, we found that tumoral and peritumoral parameters can reflect the chemotherapy response and predict EFS and overall survival, which to some extent supplement and improve the diagnostic and prognostic value of MR imaging in OS patients.

## Abbreviations

ADC: Apparent diffusion coefficient
AIF: Arterial input function
DCE: Dynamic contrast enhancement
DWI: Diffusion-weighted imaging
EFS: Event-free survival
IAUC: Initial area under the curve
Kep: Elimination rate constant
Ktrans: Capacity transfer constant
MRI: Magnetic resonance imaging
OS: Osteosarcoma
ROC: Receiver operating characteristic
ROI: Region of interest
TNR: Tumor necrosis rate
Ve: Extravascular extracellular space volume ratio
VIBE: Volumetric interpolated breath hold examination
